# Difficulty in Diagnosing Rare Cardiac Tumors: A Case Series

**DOI:** 10.7759/cureus.33367

**Published:** 2023-01-04

**Authors:** Jiaxi Dong, Ling Wang, Joseph Salvatore, Michelle W Lau

**Affiliations:** 1 Department of Geriatric Medicine, Banner Health, Phoenix, USA; 2 Department of Pathology, Phoenix VA Medical Center, Phoenix, USA; 3 Department of Hematology and Oncology, Phoenix VA Medical Center, Phoenix, USA

**Keywords:** cardiac biopsy, small cell carcinomas, spindle cell sarcoma, rare cancers, cardiac tumor

## Abstract

Primary cardiac tumors are rare because metastatic lesions from distant sites account for most masses. We are reporting two cases of malignant intracardiac masses with their diagnostic dilemma.

Our first patient is a 72-year-old male with a pertinent history of desmoplastic and spindle cell melanoma who presented after his surveillance positron emission tomography (PET) scan showed a hypermetabolic lesion in the inferior pericardium. The initial impression for this mass is recurrent malignant melanoma. After an initial negative endometrial biopsy, the patient underwent debulking surgery, and pathology revealed high-grade spindle cell sarcoma. The patient underwent chemotherapy but had a disease progression and ultimately elected hospice care.

Our second patient is a 75-year-old male with a history of stage IB adenocarcinoma of the lung who presented with progressive dyspnea. An echocardiogram revealed a moderate-sized left ventricular mass. Initial assessment based on tumor morphology and location suggested possible cardiac sarcoma. However, the patient’s subsequent cardiac biopsy revealed small cell carcinoma, likely of primary cardiac origin, as no other primary nidus of the tumor was seen. Based on this result, the patient has been started on carboplatin, etoposide, and atezolizumab and responded well as of the writing of this manuscript.

Given the rarity of malignant primary cardiac tumors and their variable clinical presentation, intracardiac masses are often diagnosed incidentally. In addition, given the high risk of biopsy for intracardiac masses, a presumptive diagnosis is rendered via imaging techniques. However, most of these tumors have no pathognomonic imaging findings, and their diagnosis relies heavily on physician interpretation and experience. Our case series illustrated the unpredictable nature of noninvasive methods and that even endometrial biopsy can return a false negative. Therefore, it is essential to be persistent in obtaining a pathological diagnosis, especially if the clinical picture is unclear. While these more invasive methods present the challenge of identifying whether the procedure is truly needed and locating a skilled operator, it could change the diagnosis entirely and open the patient up to new therapies.

## Introduction

Primary cardiac tumors are extremely rare, accounting for less than 0.1% of total cardiac tumors [1]. By contrast, metastatic lesions from the lung, esophagus, and lymphatics represent the most common source of cardiac mass [1]. We report two cases of malignant cardiac masses with their diagnostic dilemma, illustrating the importance of obtaining a pathologic diagnosis despite the inherent challenges in obtaining cardiac biopsies.

## Case presentation

Case 1

The first patient is a 72-year-old male with a history of mixed desmoplastic and spindle cell melanoma found on his left upper back in 2018. The primary tumor was excised in 2019. Sentinel lymph node biopsy at the time showed positive left axillary node involvement. The patient was treated with wide-margin re-excision followed by adjuvant immunotherapy with nivolumab for one year, ending in February 2020. His positron emission tomography (PET) scan on diagnosis showed mild tracer uptake along the inferior heart border just above the diaphragm and biopsy-proven melanoma. It was felt to reflect the misregistered physiologic activity. Computed tomography (CT) scans of the chest and abdomen during the year of adjuvant treatment reported no concerning findings. The patient remained asymptomatic from a cardiac standpoint.

**Figure 1:**
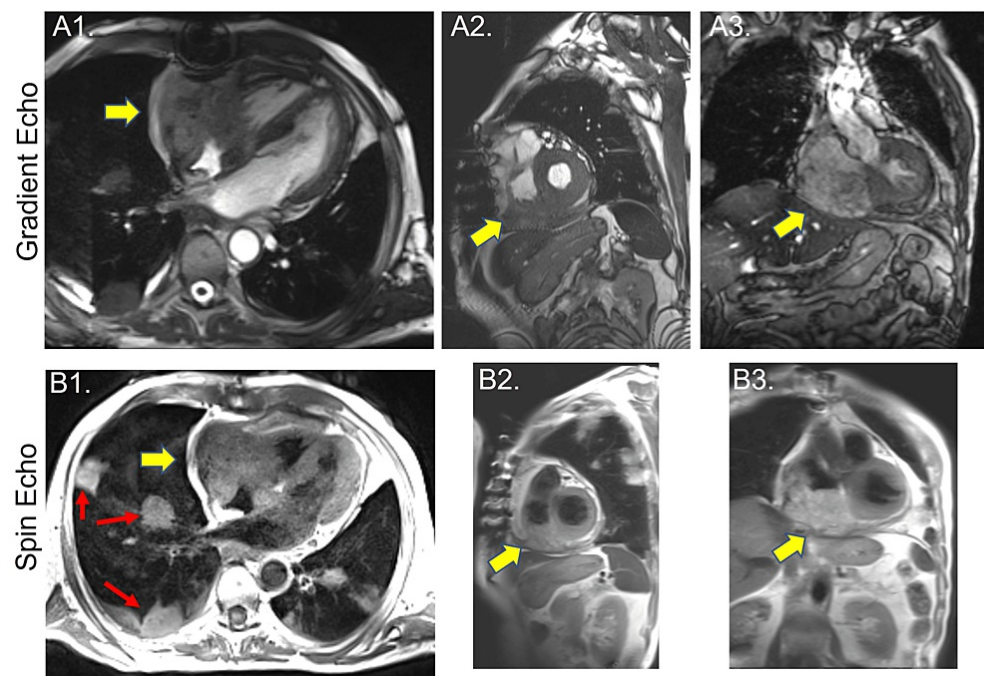
Cardiac magnetic resonance images of cardiac sarcoma Top panels show steady-state free precession gradient echo images, and bottom panels show spin echo images of a large mass in the right atrium extending to the tricuspid valve apparatus and right ventricular inflow (yellow arrows; A1 and B1 four-chamber view, A2 and B2 short axis view, A3 and B3 coronal view). Red arrows show lung masses.

A surveillance PET scan in May 2020 showed a hypermetabolic lesion in the inferior pericardium with a mass effect on bilateral atria. A cardiac magnetic resonance imaging (MRI) was performed for better visualization, which revealed a large 7.8 cm x 3.6 cm irregularly shaped mass in the inferior right atrium adjacent to the inferior vena cava and extending to the interatrial septum (Figure 1). Given the patient's clinical history, the presumptive diagnosis was metastatic malignant melanoma.

**Figure 2:**
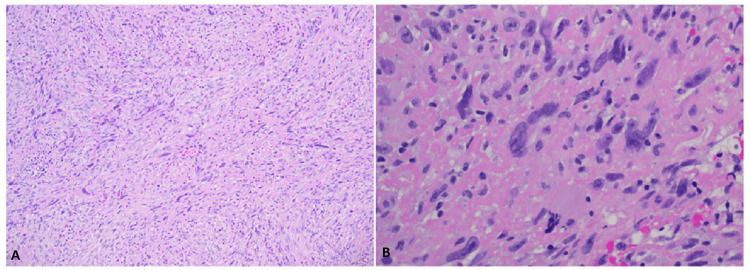
Hematoxylin and eosin (H&E) stains of the pathology sample Panel A: H&E stain shows malignant spindle cell neoplasm with vague storiform pattern, original magnification x10. Panel B: H&E stain shows malignant spindle cells with marked pleomorphism, atypical mitotic figure, and focal necrosis, original magnification x40. Histopathologically, the right atrial mass of the heart showed poorly differentiated malignant neoplasm composed of predominantly spindle cells with marked pleomorphism in a vague storiform pattern. In addition, frequent atypical mitotic figures and focal necrosis were present. Immunohistochemistry demonstrated that the malignant cells are negative for CK Oscar, SOX10, Melan-A, HMB45, S100, CD31, ERG, Desmin, smooth muscle myosin heavy chain, and myogenin. The malignant cells are positive for Vimentin and SMA (focal), considered non-specific. Overall, the findings are consistent with spindle cell sarcoma, high grade, not further classifiable.

He was initially referred to an interventional cardiologist and underwent interatrial septal mass endomyocardial biopsy (EMB), which showed no malignant cells. A repeat CT of the chest, abdomen, and pelvis in July 2020 showed no other metastatic melanoma sites. The patient was then referred to thoracic surgery for surgical biopsy and debulking. He underwent open heart surgery in October 2020, and pathology showed high-grade spindle cell sarcoma (Figure 2). The tumor's immunohistochemistry staining was only positive for vimentin, which excludes angiosarcoma, metastatic melanoma, and rhabdomyosarcoma.

He was started on gemcitabine and docetaxel, but progression was seen at imaging after three treatment cycles. He then started on proton beam radiation with gemcitabine. Post-treatment cardiac MRI in April 2021 showed stable disease. However, a repeat PET scan in July 2021 showed metastatic disease in the brain, pterygoid soft tissue, lung, and mediastinal nodes. The patient decided to pursue hospice care in July 2021 and passed away a few weeks later.

Case 2

Our second case is a 75-year-old male with a pertinent history of chronic pulmonary obstructive disease (COPD) and pulmonary nodules that were first seen on a CT scan in 2015. Video-assisted thoracoscopic surgery (VATS) was performed with wedge resection and level 4 lymph node resection in December 2016. Pathology revealed moderately differentiated adenocarcinoma with no nodal involvement (stage IB). The patient has elected surveillance at the time with no additional treatment.

**Figure 3:**
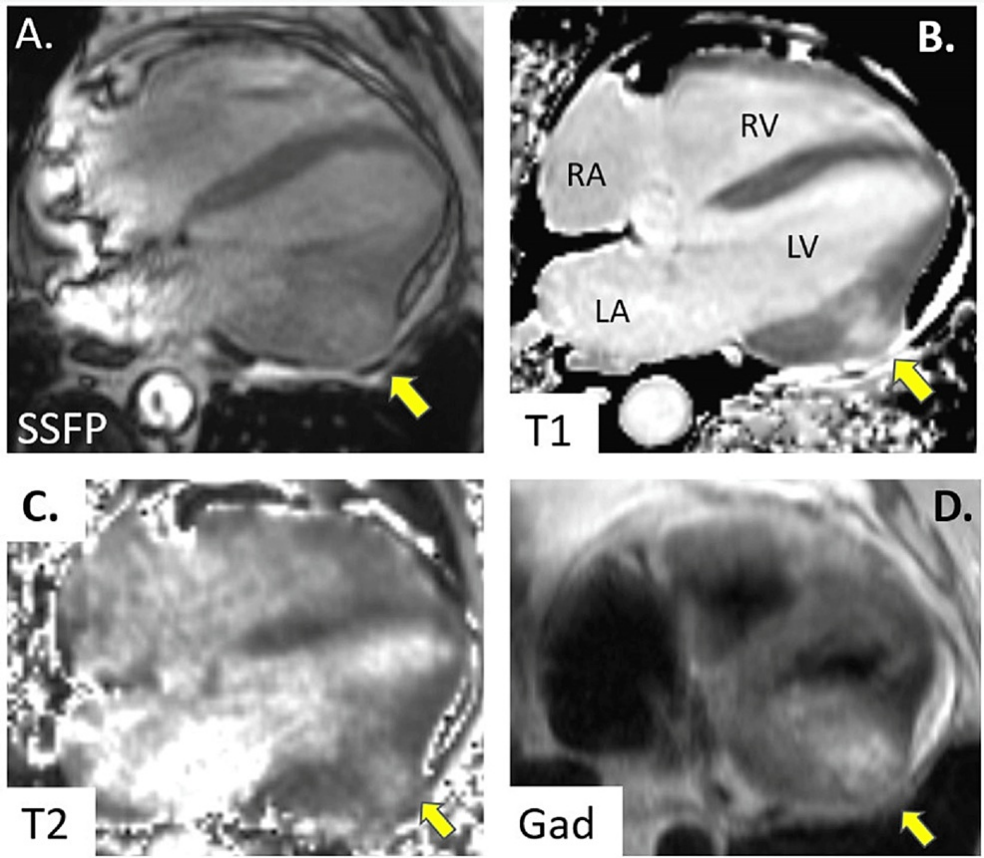
Cardiac magnetic resonance imaging Panel A: Steady-state free precession (SSFP) gradient echo image shows a bulging mass in the mid-lateral wall of the left ventricle. Panels B-D: T1 and T2 MyoMaps show increased native T1 and T2 relaxation times with late gadolinium (Gad) enhancement, suggesting increased extracellular space and edema in the mass. LV - left ventricle, RV - right ventricle, LA - left atrium, RA - right atrium

The patient presented in 2022 with acute worsening of chronic shortness of breath. He was hemodynamically stable on presentation with tachycardia and tachypnea. The physical exam was unrevealing and initial laboratories were unremarkable. A CT angiogram was done in January 2022, confirming the absence of pulmonary effusion, emboli, or consolidations. An echocardiogram was performed due to concern about heart failure, which showed an ejection fraction (EF) of 55-60%. It also incidentally revealed a moderate-sized mass in the left ventricle. A follow-up MRI showed a heterogenous mass in the mid-posterior left ventricle measuring 4.7 cm x 3.7 cm. Hypokinesia and abnormal enhancement of the associated myocardium were noted, indicating possible myocardial fibrosis, infiltration, or necrosis. A PET scan was also performed, showing hypermetabolic activity of the mass but without other signs of a tumor. No pulmonary mass was visualized on either the CT or PET scan. MRI of the brain showed a 3 mm area of enhancement in the right centrum semiovale concerning metastatic lesion, but it self-resolved on subsequent imaging (Figure 3). The initial differential diagnosis based on morphology and location was malignant cardiac sarcoma.

**Figure 4:**
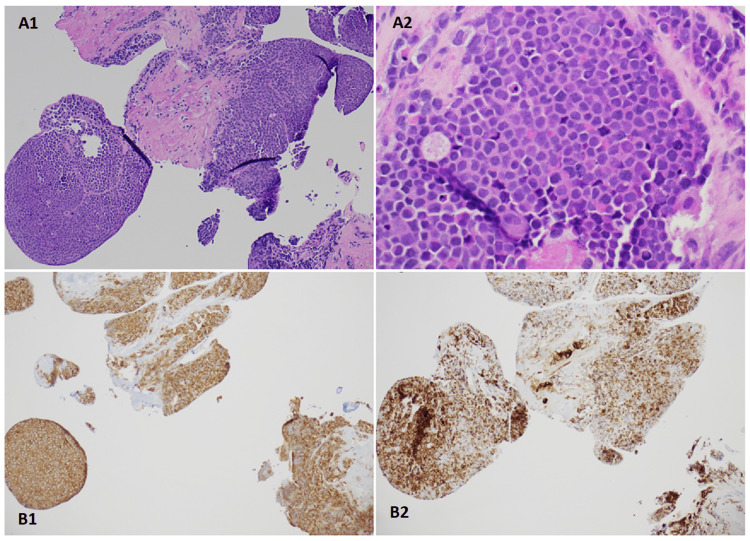
Hematoxylin and eosin (H&E) staining of the pathology sample Panel A: H&E stain shows diffuse sheets of malignant cells with scant cytoplasm, finely granular nuclear chromatin, inconspicuous nucleoli, focal crush artifact, and frequent mitoses, original magnification x10 (A1) and original magnification x40 (A2). Panel B: Malignant cells are strongly positive for Synaptophysin immunostaining, original magnification x10 (B1), and CK Oscar, original magnification x10 (B2).

The patient underwent a cardiac biopsy in March 2022, with pathology showing small cell carcinoma. The tumor was positive for CK-OSCAR, chromogranin, and synaptophysin (Figure 4). PD-L1 expression was negative.

The patient was deemed unsuitable for surgery and was started on carboplatin, etoposide, and atezolizumab soon after and is currently following up with our oncology department. The patient's repeat cardiac MRI after completion of the four cycles of chemotherapy showed a decrease in the size of the left ventricular mass. He is currently on maintenance atezolizumab immunotherapy, and his disease is stable as of September 2022.

## Discussion

Primary cardiac tumors are rare entities. It is thought that only about 0.05-1.23% of cardiac tumors are of cardiac origin [1]. About one-fourth of the primary cardiac tumors are malignant, mostly sarcomas. Nearly all subtypes of sarcoma have been reported, including angiosarcoma, rhabdomyosarcoma, fibrosarcoma, undifferentiated sarcoma, and leiomyosarcoma. These sarcomas' prognosis is often extremely poor as they could quickly proliferate and infiltrate the myocardium. In addition, metastatic diseases such as to the central nervous system (CNS) are frequent on diagnosis, limiting treatment modalities.

Metastatic disease in the heart is more common than primary cardiac malignancies, with lung cancers, gastrointestinal cancers, leukemia, and lymphomas being the most common malignancies found in the heart [1]. However, other cancers, such as melanoma, also tend to metastasize to the heart [2]. Cardiac involvement by small cell cancer, either via primary tumor or metastatic disease, is rare and only documented sporadically by several case studies [3-6]. Although in case 2, the initial pathology report stated "small cell carcinoma suspecting from pulmonary metastasis," no identifiable pulmonary primary tumor was found on either the PET scan, CT scan, or MRI of the chest. Bronchoscopy was discussed with the interventional pulmonologist and deemed low yield given all the negative imaging. Therefore we believe that his small cell carcinoma was likely a primary cardiac tumor.

As illustrated by these two cases, intracardiac masses are often found incidentally. Therefore, the work-up typically involves a follow-up cardiac MRI and a staging PET scan to delineate the mass further. Due to the high risk of biopsying a cardiac mass, a presumptive diagnosis is typically made via imaging [7]. However, there are no pathognomonic imaging findings to define cardiac mass or metastasis, except for lipoma, which could often be accurately diagnosed by CT or cardiac MRI [8].

Despite advancements in noninvasive imaging modalities, the gold standard for diagnosing cardiac tumors is by pathology. In 2007, the American College of Cardiology (ACC)/American Heart Association (AHA) published a guideline stating that EMB is reasonable for the diagnosis of cardiac tumors (class IIa recommendation) if four specific criteria are met: (1) diagnosis cannot be made in any other way, (2) the diagnosis with EMB will alter therapy, (3) the success of biopsy is believed to be reasonably high, and (4) the biopsy will be performed by an experienced operator [9]. The addition of transesophageal echocardiogram (TEE) and polymerase chain reaction (PCR) has improved the safety profile and diagnostic yield of EMB [10,11]. EMB was deemed a safe procedure, with an overall complication rate of 1-6% [9,11]. Significant complications include access site hematoma, valvular damage, pericardial tamponade, venous thrombosis, and pulmonary emboli [9]. However, our first case demonstrates that EMB can sometimes be falsely negative. In these situations, the patient often undergoes a surgical biopsy with higher risk.

For treating oncologists, the first decision is to determine whether a biopsy is needed. In case 1, our patient's recent history of melanoma led to the presumptive diagnosis of metastatic melanoma. In the absence of other metastatic foci, while the clinical suspicion of metastatic melanoma is high, it is insufficient to justify proceeding with treatment without a pathologic diagnosis. The patient's case was presented to the tumor treatment conference, and the consensus was that a biopsy was warranted. In case 2, as this patient's history of lung cancer was more remote, the need for a biopsy was initially controversial. While the multispecialty team deemed this cardiac mass highly consistent with a primary cardiac tumor, the cardiology team considered the biopsy to be of higher risk due to the need to cross the aortic valve, the possibility of transeptal puncture in a high-pressure environment, and the risk of embolism. They felt that the patient's clinical presentation and imaging results were consistent, with the mass being a primary cardiac sarcoma. Cardiothoracic surgery evaluation was that no surgical or transplant options were available for the patient; palliative care was consulted while the patient was in the hospital. The risk and benefits of proceeding with a high-risk biopsy were discussed later with the patient in the outpatient setting, and the decision was to proceed with the biopsy. The patient was referred to a university center, where a trans-catheter endomyocardial left ventricular biopsy of the left ventricular mass was performed via the antegrade trans-septal approach.

The second challenge oncologists face in diagnosing a cardiac mass is finding an experienced operator to perform the biopsy. Our hospital is located in a major metropolitan city in which there is a heart transplant center in town. Yet, since a primary heart tumor is not a common diagnosis for an oncologist, trying to identify a cardiologist to perform the procedure can be problematic. In case 1, it took us over seven weeks and multiple contacts with different cardiology and cardiothoracic surgery offices before we were directed to the university heart transplant center, where their interventional cardiologist agreed to perform the procedure. The same cardiologist group performed the biopsy on case 2. Since there are about 147 heart transplant centers and 4400 interventional cardiologists for adults in the US, community oncologists, especially those in rural areas, may have difficulty accessing the specialist needed for the procedure [12].

The third consideration is whether pathologic findings would be able to suggest new treatment options. As our two cases demonstrated, even though the prognosis is poor for cardiac tumors, finding the pathologic diagnosis did alter the patient's management. In case 2, the biopsy completely changed the direction of the patient's care. Considering that the patient was initially considered for palliative care, with pathology showing small cell carcinoma, a new treatment plan was extrapolated from metastatic small cell lung cancer. The patient's tumor stabilized after chemotherapy and remained stable on immunotherapy maintenance. In case 1, the patient desired treatment and was functional before the metastatic disease. He survived 14 months after the initial radiographic diagnosis and was stable on proton beam radiation and chemotherapy. Further complicating the decision of cardiac biopsy, as demonstrated in case 1. When EMB fails to yield a diagnosis, a surgical biopsy would be needed, which would involve an even higher risk. Since treatment only provides disease stabilization and the ultimate prognosis remains unaltered, the treatment options may not justify the risk of biopsy. This is undoubtedly a valuable question that the oncologist will need to discuss openly with their patients. Engaging palliative care for goals of care discussion may be beneficial in these situations.

## Conclusions

In conclusion, cardiac tumors are rare entities with a generally poor prognosis. However, in fit patients desiring treatment, a biopsy is needed to make the diagnosis. Although obtaining a biopsy of cardiac masses can be challenging, it may provide patients with additional treatment options otherwise not available.
